# Androgen Receptor and ALDH1 Expression Among Internationally Diverse Patient Populations

**DOI:** 10.1200/JGO.18.00056

**Published:** 2018-08-11

**Authors:** Evelyn Jiagge, Aisha Souleiman Jibril, Melissa Davis, Carlos Murga-Zamalloa, Celina G. Kleer, Kofi Gyan, George Divine, Mark Hoenerhoff, Jessica Bensenhave, Baffour Awuah, Joseph Oppong, Ernest Adjei, Barbara Salem, Kathy Toy, Sofia Merajver, Max Wicha, Lisa Newman

**Affiliations:** **Evelyn Jiagge**, **Carlos Murga-Zamalloa**, **Celina G. Kleer**, **Mark Hoenerhoff**, **Kathy Toy**, **Sofia Merajver**, **Barbara Salem**, and **Max Wicha**, University of Michigan, Ann Arbor; **George Divine**, **Jessica Bensenhaver,** Henry Ford Health System, Detroit, MI; **Evelyn Jiagge**, **Baffour Awuah**, **Joseph Oppong**, and **Ernest Adjei**, Komfo Anokye Teaching Hospital, Kumasi, Ghana; and **Aisha Souleiman Jibril**, St. Paul’s Hospital, Millenium Medical Center, Addis Ababa, Ethiopia; **Lisa Newman**, **Melissa Davis**, and **Kofi Gyan**, Weill Cornell Medicine, New York, NY.

## Abstract

**Purpose:**

Population-based incidence rates of breast cancers that are negative for estrogen receptor (ER), progesterone receptor, and human epidermal growth factor receptor 2/*neu* (triple-negative breast cancer [TNBC]) are higher among African American (AA) compared with white American (WA) women, and TNBC prevalence is elevated among selected populations of African patients. The extent to which TNBC risk is related to East African versus West African ancestry, and whether these associations extend to expression of other biomarkers, is uncertain.

**Methods:**

We used immunohistochemistry to evaluate estrogen receptor, progesterone receptor, human epidermal growth factor receptor 2/*neu*, androgen receptor and aldehyde dehydrogenase 1 (ALDH1) expression among WA (n = 153), AA (n = 76), Ethiopian (Eth)/East African (n = 90), and Ghanaian (Gh)/West African (n = 286) patients with breast cancer through an institutional review board–approved international research program.

**Results:**

Mean age at diagnosis was 43, 49, 60, and 57 years for the Eth, Gh, AA, and WA patients, respectively. TNBC frequency was higher for AA and Gh patients (41% and 54%, respectively) compared with WA and Eth patients (23% and 15%, respectively; *P* < .001) Frequency of ALDH1 positivity was higher for AA and Gh patients (32% and 36%, respectively) compared with WA and Eth patients (23% and 17%, respectively; *P* = .007). Significant differences were observed for distribution of androgen receptor positivity: 71%, 55%, 42%, and 50% for the WA, AA, Gh, and Eth patients, respectively (*P* = .008).

**Conclusion:**

Extent of African ancestry seems to be associated with particular breast cancer phenotypes. West African ancestry correlates with increased risk of TNBC and breast cancers that are positive for ALDH1.

## INTRODUCTION

Immunohistochemistry has become an essential component of breast cancer pathology, to evaluate for expression of the two hormone receptors, estrogen receptor (ER) and progesterone receptor (PR), as well as the human epidermal growth factor receptor 2 (HER2/*neu*). These three biomarkers identify patients whose disease can be manipulated with endocrine and/or targeted anti-HER2/*neu* therapies. Cancers that are negative for ER as well as PR, and that do not overexpress HER2/*neu*, are commonly referred to as triple-negative breast cancer (TNBC). Patients who are diagnosed with TNBC face a disproportionately increased risk of breast cancer mortality because of their inherently more aggressive biology and limited systemic therapy options. Population-based incidence rates of TNBC are two-fold higher in African American (AA) women compared with women with predominantly European ancestry, commonly referred to as white American (WA) women,^1,2^ and this disproportionate phenotype distribution likely contributes to breast cancer disparities, with mortality rates significantly higher among AA patients. Novel targeted therapy approaches in TNBC may involve disruption of androgenic and/or stem cell pathways. Data regarding immunohistochemistry-based measurements of proteins involved in these pathways (eg, androgen receptor [AR] and aldehyde dehydrogenase 1 [ALDH1], respectively) among diverse patient populations will therefore be valuable in the effort to use precision medicine techniques in addressing breast cancer disparities. Existing data raise more questions than answers regarding suspected associations between African ancestry, TNBC, and breast cancer stem cell biology.^3^ Prior studies suggest variation in phenotype distribution related to West versus East African ancestry,^4,5^ and we therefore sought to compare patterns of this broader spectrum of tumor markers in AA and WA patients as well as in patients from either coast of Africa.

## METHODS

We evaluated ER, PR, HER2/*neu*, AR, and the mammary stem cell marker ALDH1 by immunohistochemistry analysis on formalin-fixed, paraffin-embedded invasive breast cancer specimens from a Michigan-based international biorepository, the Henry Ford Health System International Center for the Study of Breast Cancer Subtypes. The cases analyzed represented a selection of female patients with breast cancer with four different backgrounds evaluated between 2000 and 2014: AA, WA, Ghanaian/West African (Gh), and Ethiopian/East African (Eth). AA and WA patients were treated at the University of Michigan Comprehensive Cancer Center and were categorized by self-reported racial/ethnic identity; Gh and Eth patients were native to and residing in those countries. All specimens were collected through convenience sampling of tissues available from patients receiving treatment at the retrospective institutions. Because of limited medical record-keeping capacity at the African hospitals, no information was consistently available regarding clinical aspects of disease beyond patient age (eg, menopausal status, parity, clinical stage, clinical outcomes, diagnostic and/or treatment details).

This work was approved by the institutional review boards and human ethics equivalents of the University of Michigan, the Henry Ford Health System, the Komfo Anokye Teaching Hospital in Kumasi, Ghana, and the Millennium Medical College St. Paul’s Hospital in Addis Ababa, Ethiopia. Immunohistochemistry for all five biomarkers was performed and interpreted by pathologists at the Henry Ford Health System and the University of Michigan.

### Pathology and Immunohistochemistry

Histopathology assessment on paraffin-embedded sections stained with hematoxylin and eosin was performed to confirm the diagnosis. Immunohistochemistry was performed with the streptavidin-biotin immunoperoxidase method at the departments of pathology at the Henry Ford Health System and the University of Michigan North Campus Research Complex. Immunohistochemistry for ER and PR was performed with monoclonal mouse antibodies to human ER (DAKO clone ID5; DAKO, Glostrup, Denmark) and to human PR (DAKO clone PgR636). Tumors were scored as ER/PR-positive if they feature more than 1% nuclear staining. Immunohistochemistry for HER2/neu staining was performed using the HerceptTest (DAKO). Grading of HER2 expression was based on recommendations from Fitzgibbons et al.^6^ Any specimen scored as 0 or 1+ was classified as HER2/neu negative, and specimens scored as 3+ were considered positive. Specimens with a score of 2+ were considered equivocal, and follow-up fluorescent in situ hybridization was used to assess amplification of the *HER2/neu* gene. Fluorescent in situ hybridization for *HER2/neu* gene amplification was interpreted in compliance with ASCO/College of American Pathologists guidelines.^6-8^ Tumors that were negative for ER, PR, and HER2/*neu* were classified as TNBC. Immunohistochemistry for ALDH1 was performed with mouse monoclonal antibodies (BD Biosciences, San Jose CA; clone 44). Expression of ALDH1 was scored as positive if more than 5% of cells showed cytoplasmic stain, as described.^9^ Immunohistochemistry for AR was performed with rabbit monoclonal antibodies (Cell Marque, Rocklin, CA; clone SP107). AR expression was scored as positive if more than 10% of tumor cells show nuclear staining, as described.^10^ These results were interpreted by three pathologists (C.G.K., M.H., and C.M.Z.), who evaluated the Gh and Eth cases as de-identified/anonymized specimens but had access to patient identifying information for the AA and WA cases.

Statistical analyses were performed using SAS 9.1 (SAS Institute, Cary, NC). Categorical variables were compared by χ^2^ analysis, and continuous variables were compared by Student *t* test.

## RESULTS

Table 1 summarizes the clinicopathologic features of the WA, AA, Gh, and Eth patients. The two American patient subsets were significantly younger than the African patients (median ages, 56.8 and 60.2 years, respectively, *v* 49.3 and 42.7 years, respectively; *P* < .001).

**Table 1:**
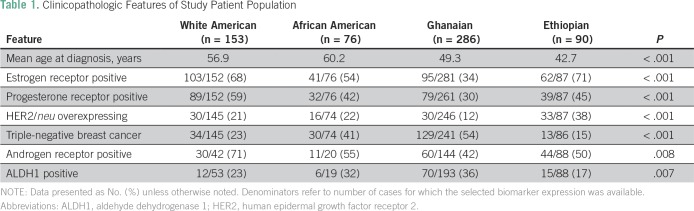
Clinicopathologic Features of Study Patient Population

Frequency of ER-positive disease was higher in the WA and Eth patients (68% and 71%, respectively) than in AA and Gh patients (54% and 34%, respectively), and the differences across this distribution were statistically significant (*P* < .001). Similarly, frequency of TNBC was increased among AA and Gh patients (41% and 54%, respectively) compared with WA and Eth patients (23% and 15%, respectively), another statistically significant distribution (*P* < .001). HER2/neu-overexpressing cancers (109 of 552; 19.7%) were less prevalent among WA, AA, and Gh patients (21%, 22%, and 12%, respectively) than Eth patients (38%; *P* < .001).

Frequency of ALDH1 positivity was also higher for the AA and Gh tumors (32% and 36%, respectively) compared with the WA and Eth tumors (23% and 17%, respectively; *P* = .007). Prevalence of AR positivity was increased among the WA patients (71%) compared with all three of the African ancestry populations (55%, 42%, and 50%, for the AA, Gh, and Eth patients, respectively; *P* = .008).

As shown in Tables 2 and 3, these patterns persisted after stratifying by age younger than 50 years and age older than 50 years (age was not confirmed for two WA, three AA, and one Gh patient). Regardless of age category, the AA and Gh patients were more likely to have ER-negative breast cancer and TNBC than the WA and Eth patients; the Eth patients were more likely to have HER2/neu-overexpressing tumors. Statistical comparisons were less stable for the age-based subset analyses because of the relatively smaller number of cases with complete biomarker information available, but the trends persisted for WA patients having the highest prevalence of AR-positive tumors and for Gh patients having the highest prevalence of ALDH1-positive tumors (Table 4).

**Table 2:**
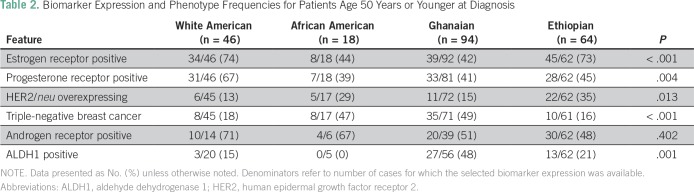
Biomarker Expression and Phenotype Frequencies for Patients Age 50 Years or Younger at Diagnosis

**Table 3:**
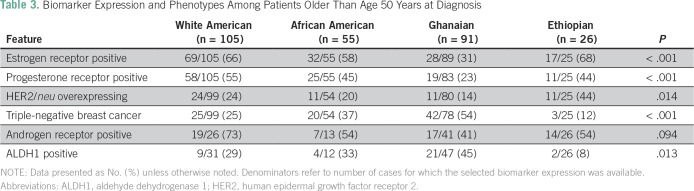
Biomarker Expression and Phenotypes Among Patients Older Than Age 50 Years at Diagnosis

**Table 4:**
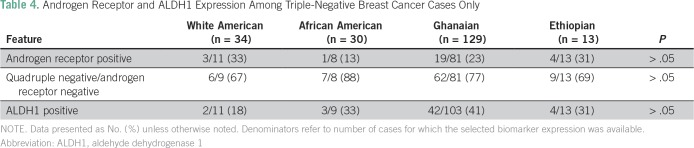
Androgen Receptor and ALDH1 Expression Among Triple-Negative Breast Cancer Cases Only

## DISCUSSION

Disparities in breast cancer burden related to racial/ethnic identity have been documented for several decades, with AA patients having a more advanced stage distribution and higher mortality rates compared with WA patients.^11^ Metrics of socioeconomic status, such as poverty rates and lack of health care insurance, are also higher in the AA community, and these socioeconomic status disadvantages undoubtedly contribute to breast cancer outcome differences by causing diagnostic as well as treatment delays.^12^ Several investigators have nonetheless speculated that primary differences in breast tumor biology associated with AA identity might also exist.^11,13,14^

Indeed, it is now well established that population-based incidence rates of the biologically aggressive TNBC phenotype is approximately two-fold higher in the AA compared with WA community.^1^ TNBC actually comprises a diverse spectrum of tumor subsets, but approximately three quarters belong to the inherently virulent basal subtype; this association between TNBC and AA identity therefore also plays a significant role in explaining breast cancer disparities.^15^ Furthermore, TNBC identifies patients who are more likely to have hereditary susceptibility for cancer related to germline *BRCA1* mutations.^16^ This constellation of correlations prompts questions regarding whether African ancestry is an independent marker of risk for biologically aggressive breast cancer patterns.

Africa is a large continent, associated with significant genetic and cultural heterogeneity. Individuals who self-report as having AA background have predominantly shared ancestry with western, sub-Saharan Africa, a consequence of the trans-Atlantic slave trade. Ghana is located in this region of Africa, making this country well suited for comparisons of breast tumor phenotypes in women with varying degrees of West African heritage. In contrast, the east African slave trade was largely controlled by Arabic traders and resulted in forced migration of east Africans to the Mideast and to Asia. Self-reported AA individuals in the United States therefore have less shared ancestry with East Africans, including Ethiopians.^11^

Our international breast cancer research program has previously demonstrated that the distribution of breast cancer phenotypes is comparable for African Americans and Ghanaians, with regard to an increased prevalence of TNBC.^17,18^ In contrast, the frequency of TNBC is similarly low for Eth and WA patients.^4^ Data on TNBC rates in Ethiopia are sparse, but the relatively low frequency of ER-negative breast cancer was also shown by Kantelhardt et al.^19^ Interestingly, Jemal and Fedewa^5^ analyzed data from the SEER program to compare frequencies of ER-negative breast cancer among AA and WA women, as well as among women born in either East Africa or West Africa, but who developed breast cancer in the United States. Similar to our findings regarding TNBC, AA and West Africans had relatively higher frequencies of ER-negative disease compared with WA and East African patients, where the rates of ER-negative tumors were relatively low.

The current study expands on our group’s earlier work. In addition to using immunohistochemistry to evaluate ER, PR, and HER2/*neu* expression, we also report on expression of AR and ALDH1. We chose these two additional biomarkers because of their potential roles in TNBC pathogenesis and management. TNBC is now known to include a diverse spectrum of subtypes identified by gene expression studies.^20,21^ The luminal androgen receptor subtype tends to respond poorly to neoadjuvant chemotherapy^22^ and may represent a pattern that can be manipulated with targeted antiandrogen therapy.^23-25^ Immunohistochemistry to assess AR expression may therefore have value in TNBC treatment planning. Although there are inconsistent findings in the reported literature, ALDH1 has been proposed as a marker of the mammary stem cell and TNBC virulence.^26-28^ We and others have previously reported on elevated expression of ALDH1 in Ghanaian^29^ and Ugandan patients.^30^

We found that all three African ancestry population subsets had relatively lower frequencies of AR expression compared with WA (ranging from 42% to 55% *v* 71%), but otherwise the frequencies of the various markers and phenotypes demonstrated similarities between AA and Gh patients with breast cancer; conversely, the WA and Eth patients with breast cancer were more similar to each other.

Our study has several important limitations. For US-based WA and AA patients, early detection results in smaller tissue samples available for research studies, and therefore many patients could not be evaluated for AR and ALDH1. Unfortunately, the financial constraints of the Ghanaian and Ethiopian participating facilities precluded consistent availability of detailed medical records regarding reproductive/gynecologic and family history. We therefore had to rely on age at diagnosis, which was routinely recorded at all sites. Although the pathology processing of the US specimens was standardized in accordance with institutional and professional guidelines for prompt handling during the 2000 to 2014 study period, the Ghanaian and Ethiopian sites did not have resources to implement comparable standards. We expect, however, that the similar financial constraints present in the Ghanaian and Ethiopian sites would not have explained the divergent phenotype distributions observed between patients from these two sites. Also, the convenience-based nature of our sample assembly could have interjected biases that are not necessarily obvious: the Ethiopian cases were all based on samples retrieved from surgical resections (because of limited availability of diagnostic needle biopsy technology), whereas the US and Ghanaian cases represented a combination of surgical specimens and core needle biopsy specimens. Last, given the well-documented association between TNBC and hereditary susceptibility for breast cancer via BRCA1 mutation carrier status, germline genetic testing would have potentially yielded meaningful comparative results in our study population subsets. Unfortunately, however, neither genetic counseling nor genetic testing is routinely available in Ghana and Ethiopia, so this information was not available. We also hope that future international collaborative research efforts will include accurate data on patient follow-up, so that outcomes can be assessed.

These findings are hypothesis generating and support the need for additional research regarding associations between African ancestry and TNBC. The genotyping technology of ancestry-informative markers is a promising strategy that can discern East versus West African heritage and may be particularly helpful in understanding breast cancer risk related to heritage among admixed populations. Thus, application of germline genomics may assist in understanding the influence of geographically defined ancestry on breast cancer risk.^31^
